# Redundancy in Genotyping Arrays

**DOI:** 10.1371/journal.pone.0000287

**Published:** 2007-03-14

**Authors:** Scott Smemo, Justin O. Borevitz

**Affiliations:** 1 Department of Human Genetics, University of Chicago, Chicago, Illinois, United States of America; 2 Department of Ecology and Evolution, University of Chicago, Chicago, Illinois, United States of America; Indiana University, United States of America

## Abstract

Despite their unprecedented density, current SNP genotyping arrays contain large amounts of redundancy, with up to 40 oligonucleotide features used to query each SNP. By using publicly available reference genotype data from the International HapMap, we show that 93.6% sensitivity at <5% false positive rate can be obtained with only four probes per SNP, compared with 98.3% with the full data set. Removal of this redundancy will allow for more comprehensive whole-genome association studies with increased SNP density and larger sample sizes.

## Introduction

High density genotyping arrays have been heralded as one of the technologies that will enable whole-genome association tests to make good on the promise that sequencing the human genome will reveal the genetic causes of complex diseases [1]–[4]. While currently-available arrays, or chips, have the capacity for assaying hundreds of thousands of Single Nucleotide Polymorphisms (SNPs) in a single experiment, data from millions of SNPs across a large number of samples is needed to fine map complex traits. Fortunately, with current technology, this is already possible because available arrays have a large amount of redundancy—multiple oligonucleotide features (probes) are used to assay the same SNP. If that redundancy were reduced or even eliminated, commensurately more data could be obtained for the same price. We therefore sought to determine the minimum number of features necessary to accurately assign genotypes to Affymetrix's GeneChip.

## Materials and Methods

The physical Affymetrix GeneChip Human Mapping 100K chip contains 116,204 biallelic SNPs interrogated in two subsets that differ in which restriction enzyme was used in their preparation, either XbaI or HindIII [5]. We analyzed only the Xba subset but assume the results will be applicable to the Hind data. The Affymetrix GeneChip Human Mapping 100K data set (http://www.affymetrix.com/support/technical/sample_data/hapmap_trio_data.affx) contains raw, feature-level data for 90 CEPH individuals genotyped across these 116,204 SNPs. Our analyses are based on the Xba subset, which contains 58,960 SNPs. Most of these (58,011, 98%) were also included in the HapMap Project [6]. HapMap SNP genotypes were used as our gold standard because of the project's quality control measures (trios for genotyping, multi-site verification), though we and others [7] acknowledge errors may still be present in this data. The Affymetrix data set lacked genotype calls for 4,651 SNPs and were omitted in all subsequent analyses, leaving 53,360 SNPs in our working set.

We used the Bioconductor [8] package Robust Linear Model with Mahalanobis Distance Classifier (RLMM) [7] for making genotype classifications or calls. By using a multi-chip, multi-SNP approach, predicated on a large training set, RLMM has been shown to be an accurate and informationally efficient method [7]. A variation, Bayesian RLMM, is now provided by Affymetrix for analyzing their 500K arrays as a white paper (BRLMM, http://www.affymetrix.com/support/technical/product_updates/brlmm_algorithm.affx), and an improved method, CRLMM, was just released as an R/oligo developmental package [9].

**Figure 1:**
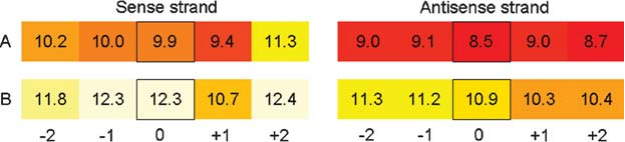
Probe set layout for Affymetrix 100K chip. Each SNP is assayed with 20 perfect match 25-mer oligonucleotides. The additional 20 mismatch oligonucleotides are not shown. Both sense and antisense strands are interrogated with 5 features for each allele that differ only at the SNP nucleotide, denoted A and B. The position of the SNP within the 25mer features is shifted from the central nucleotide (boxed, “0”) by 1 or 2 nucleotides in either direction. For this SNP (rs836702), and sample (NA11994) allele B has a stronger normalized hybridization signal, as indicated by the lighter color and higher hybridization values for most probes. Generally the central base and a particular strand have more discrimination specificity. The genotype for this individual was called as BB with full and all reduced probesets. This is clear when many other samples are tested such that the range of intensity categories is known.

The Affymetrix arrays contain probe sets for each SNP with up to 20 perfect match and 20 mismatch features (Fig. 1). RLMM, BRLMM, CRLMM do not use mismatch data, making ½ of the array immediately redundant. We modified RLMM to allow genotype assignments to be made using specific subsets of features. This includes all 5 feature positions (the default for RLMM with the 100K chip), the 3 central-most (default for the 500K chip), and a single central feature position. By default RLMM uses the information from both the sense and antisense strands. We further modified it to allow for selection of only a single strand using two different methods. One method selects the strand with the strongest average signal across features of both A and B alleles, while the other method uses the strand with the maximal allele intensity difference. These 6 parameter combinations (1, 3, or 5 features per strand; 1 or 2 strands) were used in turn to make genotype classifications, with a 100% calling rate setting in RLMM. It is also possible to use a single probe for genotyping using intensity alone to make the call. This approach called Single Feature Polymorphism [10] was not investigated here as both alleles are known and included on the array. Additional workflow information, including our full data sets and algorithms, are available online (http://www.naturalvariation.org/snpredundancy).

## Results

We first tested for internal concordance in genotype classification, comparing genotype calls made with reduced probesets to the full probeset of 20 features (5 positions, 2 strands, 2 alleles). For each SNP, the number of discordant genotypes was counted. That is, for each of the 90 individuals in the data set, their reduced probeset-derived genotype was compared to the full probeset-derived genotype. Supplemental Figure 1 and Supplementary Table S1 show the cumulative distribution of SNPs at different discordance thresholds. Notably, even with only 2 features (1 central position & 1 strand, or 10% of the total data), half of the SNPs are called in perfect concordance and 78% have 2 or fewer discordant calls. When both strands are used, 66% of SNPs have perfect concordance and 85% have 2 or fewer discordances.

**Figure 2:**
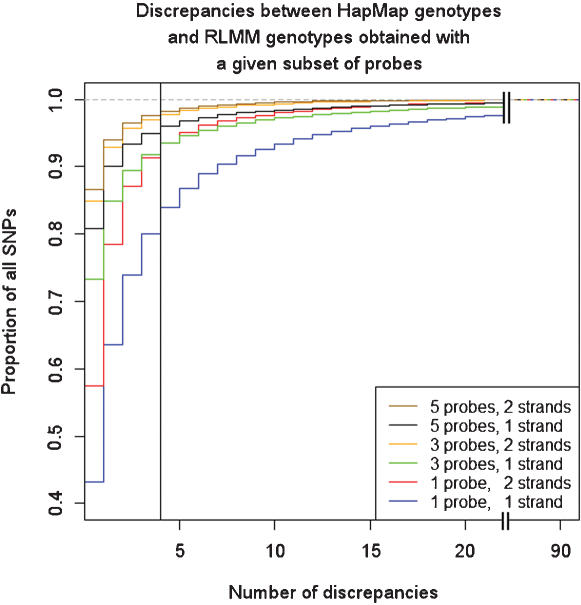
Cumulative distribution of discrepancies between HapMap and RLMM genotypes across probe set combinations Each curve shows the cumulative proportion of discrepancies between the 90 individuals for which genotypes were called using the indicated RLMM reduced probeset compared against the HapMap project. The vertical line at 4 discrepancies corresponds to approximately 95% specificity. 93.6% of all SNPs had 4 or fewer discrepancies when only the central probe on both strands was used (red curve). Note the break at 30 discrepancies, at which point 5 of the 6 curves have all but converged.

We next tested specificity and sensitivity with independent genotype classification by comparing RLMM genotype calls made with various feature subsets to HapMap genotypes. Again, for each SNP, specificity was measured as the number of discrepancies across samples (Figure 2, x axis). Sensitivity is shown as the proportion of SNPs with given specificity. The full model is less than perfect and achieves 98.3% sensitivity at 95% specificity (4/90 or fewer discrepancies). So, how much worse are less redundant designs? The reduced models with 1 or 3 features on 2 strands perform quite well achieving 93.6% and 96.8%, respectively. Figure 2 shows the cumulative distribution of discrepancies across all SNPs. The single strand with maximum intensity was also quite predictive and would be important for RNA assays. 3 positions on 1 strand (6 features) was better than 1 position on 2 strands (4 features), and 5 positions on 1 strand (10 features) was worse than 3 positions on 2 strands (12 features), as would be expected given extra redundancy (Supplemental table S2). The point is that the improvement with extra data is minimal.

We next considered the manner in which discrepancies occurred by comparing how genotypes should have been called (HapMap AA, AB, BB calls) to how they were actually called (RLMM AA, AB, BB calls) for each of the reduced probesets. As has been previously reported [7], heterozygotes were most prone to misclassification. For example SNPs typed at 1 position across two strands, hets are miscalled 2.8% and homozygotes each at 0.4%. This bias however, was not greatly exaggerated for the reduced probesets (Supplementary Table S3).

**Figure 3:**
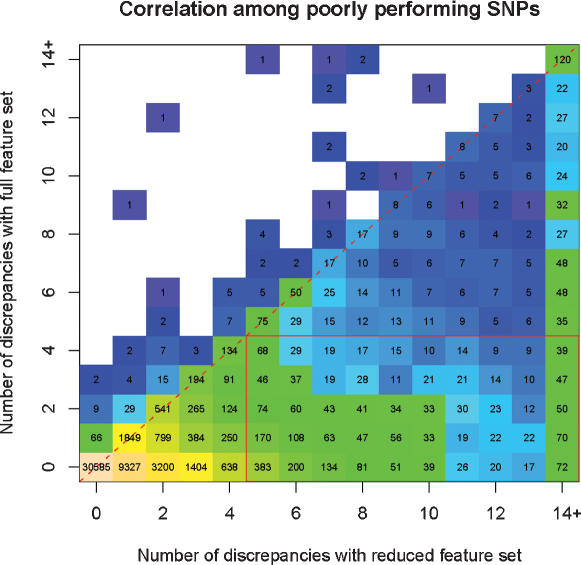
Correlation among poorly performing SNPs The number of discrepancies between HapMap calls and RLMM calls made with the reduced or full feature genotype data. Most SNPs (30,585) are perfectly called with both the full and reduced sets (peach), while another 3,030 poor SNPs fall along the diagonal and are not improved with data from additional features. Those few 2,516 SNPs (red box) that do improve with additional features do not justify redundant designs for all SNPs on the array.

Finally, we tested if the genotyping errors were non-randomly distributed, perhaps due to bad samples or to bad SNPs. No DNA sample gave an abnormally large number of discrepant genotypes as would be the case with a poor sample or array; however some SNPs performed poorly, independent of the redundancy in the probeset. For example, we compared genotyping discrepancies for genotypes generated using a reduced probeset (1 position, 2 strands, 2 alleles) to the full probeset (5 positions, 2 strands, 2 alleles) in Figure 3. 33,712 SNPs (63%) lie on or above the diagonal and are not improved by the full feature set. The majority of these, 30,585 SNPs, are called perfectly in either case. 2,516 SNPs benefit from larger probesets and do pass (<%5 false positives) with the full probeset. However, more data does not always help: 17 SNPs that fail (>5% false positives) with the full set actually pass with the reduced set.

In general, poorly performing SNPs reduce the overall performance of the array and should be eliminated in future array designs. For example, by removing SNPs that had more than 1 discrepancy when using the full feature set (6.04% of all SNPs), the overall proportion of SNPs with 4 or fewer discrepancies (5% false positive) when typed with 2 strands 1 and position increased by 3.15% to 96.7%.

## Discussion

We have shown that genotypes classified with as few as 4 features are nearly as sensitive as those classified using sets with 20 features (93.6% vs 98.3% at a 95% specificity threshold). While additional features generally improve sensitivity and specificity, they do so at a diminishing rate. The discrepancy rate for SNP genotypes was correlated between full and reduced probeset calls, indicating poorly performing SNPs are only slightly improved with additional features. By contrast, 58% of SNPs are perfectly genotyped with only 4 features improving to 87% with the full set. This improvement comes at a cost of 5–10× in SNP real estate. By thoughtfully choosing which SNPs to include, and drastically reducing the number of features to use for each SNP, large increases in total SNPs can be realized in new array designs. At the current 5 µm feature density, GeneChip arrays contain 6.55 million features. At 4 features per SNP >1.5 million SNPs can be achieved. In addition, as more robust genotype calling methods are developed (BRLMM, CRLMM) [9], accuracy will increase, further reducing any necessity for feature redundancy. Together this will allow a single genotyping array to have SNP densities high enough to enable true genome-wide association studies in the near future.

## Supporting Information

Figure S1 Cumulative distribution of internal discordances across probeset combinations Each curve shows the cumulative proportion of discrepant genotypes obtained when genotypes are called using the full probe set compared to genotyped called using the indicated subset of probes. While the total number of discrepancies is out of a possible 90, only the first 30 are shown, which is the point at which the 5 curves converge.
(0.63 MB TIF)

Table S1 Cumulative distribution showing the proportion of SNPs typed with a maximum number of discrepencies when compared to genotypes generated with all 5 probes and both strands.By default RLMM uses the information from both the sense and antisense strands, but we further modified it in two ways. Method 1 selected the strand with the strongest signal averaged across A and B probe sets. Method 2 selected the strand with the larger difference between average A and average B allele intensities. Performance for method 2 was inferior across all parameter combinations, therefore method 1 results are shown in subsequent figures/tables.
(0.02 MB XLS)

Table S2 Cumulative distribution showing the proportion of SNPs typed with a maximum number of discrepencies when compared to HapMap genotypes.By default RLMM uses the information from both the sense and antisense strands, but we further modified it in two ways. Method 1 selected the strand with the strongest signal averaged across A and B probe sets. Method 2 selected the strand with the larger difference between average A and average B allele intensities. Performance for method 2 was inferior across all parameter combinations, therefore method 1 results are shown in subsequent figures/tables.
(0.02 MB XLS)

Table S3 Misclassified genotypes accumulate in heterozygotes.The number of probes per strand and strands used is indicated in the upper left-hand corner of each box. The numbers represent counts of genotypes binned by their “true” genotypes (HapMap calls) compared to how RLMM classified them (RLMM Calls). When only one probe and one strand are used, a large excess of misclassified heterozygotes is seen. False positive rate corresponds to the proportion of misclassified genotypes in each column or row. Relative amount indicates the proportion of each type of false positive within the total number of misclassified genotypes.
(0.02 MB XLS)
