# Supplementary figures and images for: Redundancy in Genotyping Arrays
Source: PLoS One. 2007 Mar 14;2(3):e287. doi: 10.1371/journal.pone.0000287 (PMC1805688; doi:10.1371/journal.pone.0000287)

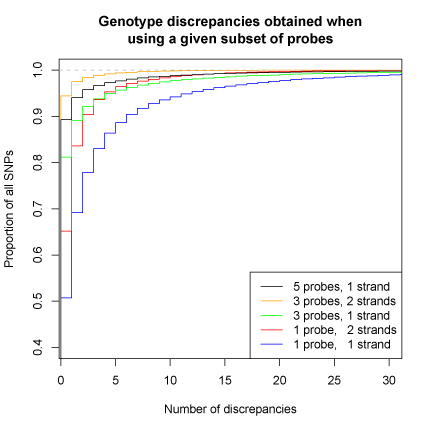

Supplement: Figure S1 — Cumulative distribution of internal discordances across probeset combinations Each curve shows the cumulative proportion of discrepant genotypes obtained when genotypes are called using the full probe set compared to genotyped called using the indicated subset of probes. While the total number of discrepancies is out of a possible 90, only the first 30 are shown, which is the point at which the 5 curves converge. (0.63 MB TIF) [file pone.0000287.s001.tif]
